# Spoofing Attack on Ultrasonic Distance Sensors Using a Continuous Signal

**DOI:** 10.3390/s20216157

**Published:** 2020-10-29

**Authors:** Tomer Gluck, Moshe Kravchik, Samuel Chocron, Yuval Elovici, Asaf Shabtai

**Affiliations:** 1 Department of Software and Information Systems Engineering, Ben-Gurion University of the Negev, Beer-Sheva 8410501, Israel; gluckt@post.bgu.ac.il (T.G.); moshekr@post.bgu.ac.il (M.K.); elovici@bgu.ac.il (Y.E.); 2 Rafael Advanced Defense Systems Ltd., Haifa 3102102, Israel; SAMUELC@rafael.co.il

**Keywords:** autonomous vehicles, drones, sensors, ultrasonic sensor, sensors attack, spoofing

## Abstract

Ultrasonic distance sensors use an ultrasonic pulse’s time of flight to calculate the distance to the reflecting object. Widely used in industry, these sensors are an important component in autonomous vehicles, where they are used for such tasks as object avoidance and altitude measurement. The proper operation of such autonomous vehicles relies on sensor measurements; therefore, an adversary that has the ability to undermine the sensor’s reliability can pose a major risk to the vehicle. Previous attempts to alter the measurements of this sensor using an external signal succeeded in performing a denial-of-service (DoS) attack, in which the sensor’s reading showed a constant value, and a spoofing attack, in which the attacker could control the measurement to some extent. However, these attacks require precise knowledge of the sensor and its operation (e.g., timing of the ultrasonic pulse sent by the sensor). In this paper, we present an attack on ultrasonic distance sensors in which the measured distance can be altered (i.e., spoofing attack). The attack exploits a vulnerability discovered in the ultrasonic sensor’s receiver that results in a fake pulse that is produced by a constant noise in the input. A major advantage of the proposed attack is that, unlike previous attacks, a constant signal is used, and therefore, no prior knowledge of the sensor’s relative location or its timing behavior is required. We demonstrate the attack in both a lab setup (testbed) and a real setup involving a drone to demonstrate its feasibility. Our experiments show that the attack can interfere with the proper operation of the vehicle. In addition to the risk that the attack poses to autonomous vehicles, it can also be used as an effective defensive tool for restricting the movement of unauthorized autonomous vehicles within a protected area.

## 1. Introduction

Ultrasonic distance sensors (UDSs) are active sensors that can provide accurate distance readings for relatively large objects in the range of around two centimeters to five meters [1]. The UDS operates by sending a pulse of ultrasonic sound waves and calculating the distance to a nearby object, using the time of flight of the returning signal (i.e., the echo). The UDS is characterized by its moderate refresh speed, short distance, and low-resolution sensor. It is slower than other electromagnetic-based distance sensors, as it is limited to the speed of sound. In addition, the fact that sound cannot travel efficiently for long distances limits its distance reading capability to a few meters. The UDS sends a pulse in the direction of the scanned area, and if the echo is strong enough, it will be interpreted as an object; hence, these sensors can only detect relatively large objects.

Ultrasonic sensors are found in different types of autonomous vehicles and assist in performing various tasks. In cars, they are commonly used for parking assistance (to notify the user when the car is in close proximity to nearby objects) [2] and navigating around nearby obstacles in autonomous driving [3]. In drones (and other robotic platforms), UDSs are used for collision avoidance and safe landing.

As cars, drones, and other vehicles increasingly rely on these sensors, the safety of these platforms has become a major concern. Consequently, protecting these platforms against sensor-based attacks is essential. Sensor attacks can impact the safety and reliability of an autonomous vehicle, where a compromised sensor can affect the vehicle’s stability and can result in damage to the vehicle or physical harm to the surrounding environment. However, the ability to alter the sensor’s operation is relevant from both the offensive and defensive perspectives. Defending against unauthorized/hostile autonomous vehicles and preventing them from approaching a sensitive facility (e.g., for espionage or terrorist attacks) is highly important [4,5]. As a case in point, in 2017, South Carolina authorities reported that (presumably) an inmate escaped using tools that were delivered to him by a hobbyist drone https://www.nytimes.com/2017/07/07/us/drone-inmate-escape.html.

Previous attacks on ultrasonic sensors have demonstrated the ability to neutralize the sensor or control its readings [6]. Controlling the sensor is the more harmful type of attack, as it can cause more significant and unexpected damage. These attacks, however, are expensive, involve a complex setup, and are difficult to execute; therefore, these attacks have mainly been demonstrated in closed and controlled environments and are difficult to replicate in real setups.

In this research, we present and evaluate a novel spoofing attack that enables the attacker to control the readings of the sensor by changing the amplitude or frequency of a continuous sound signal generated by the attacker. Unlike previous attacks, the proposed attack is simple to implement and can be executed using inexpensive components. We successfully demonstrate the attack in a lab setup (testbed) consisting of commonly used ultrasonic distance sensors and basic hardware. We also tested the attack on a real drone (DJI Mavic Pro) equipped with ultrasonic distance sensors that support its autonomous and safe landing features and demonstrate the attack’s effectiveness in different scenarios, including a scenario that may result in physical damage to the drone.

The rest of the paper is organized as follows: In Section 2, we present recent related works that focus on attacks on ultrasonic distance sensors. In Section 3, we present our proposed attack, our analysis of the root cause of the attack (i.e., the vulnerability that allows the implementation of the attack, as well as a formal calculation for the number of transmitters needed to attack a sensor from a given distance). In Section 4, we present our experiments and implementation of the attack on a testbed, as well as on a real drone. We evaluate the attack transferability (i.e., the ability to determine the attack properties on one sensor and replicate it with the derived parameters on other sensors of the same model) and robustness (i.e., applicability of the attack on different sensors). In Section 5, we present several possible countermeasures for mitigating the attack, and Section 6 concludes the work and presents possible future work.

## 2. Related Work on Attacks on Ultrasonic Distance Sensors

There are several known attacks on the ultrasonic distance sensor that demonstrate the ability to disable it or control its readings, starting with the work by Yan et al. [6]. Some of the known attacks have realistic case scenarios in which they can be executed, some have to be implemented in a controlled setup, and some are possible in theory but have not yet been implemented in a real setup. Recently, Yan et al. suggested a formalization of analog sensor security and a classification of the attacks [7]. Based on this classification, in Table 1, we categorize the previously published attacks on ultrasonic sensors as denial-of-service, spoofing, acoustic cancellation, and adversarial learning attacks, and we elaborate on each of these methods below.

**Denial-of-service (Jamming).** In this case, by creating continuous interference, the attacker can force the sensor to infer an incorrect distance, as demonstrated by Yan et al. [6] on a number of car models. Depending on the way in which the sensor operates, two possible scenarios can occur with continuous jamming: simulating the minimum distance that might, for example, cause the car to stop or an infinite or maximum distance that will prevent it from stopping, as demonstrated by Xu et al. [9] with Tesla Model S. The minimum distance scenario is possible with sensors that use a constant threshold for echo validation; in this case, the moment that the sensor starts listening, the jamming wave triggers the return echo, and the minimum distance will be derived. The infinite distance scenario is possible with sensors that measure the noise floor of the surrounding area, and the threshold is set accordingly; in this case, if the jamming noise is strong enough, the echo cannot overcome the high threshold, and the sensor will not receive an echo. Another form of a DoS attack involves physically blocking the sensor or manipulating the environment, such that the echo will not be received, as shown by Lim et al. [8] in a laboratory setup. The blocking can be performed by covering objects with foam or other material with similar properties that reduce the echo, thus making the object invisible to the sensor.
**Spoofing.** By timing a fake echo pulse when the sensor is waiting for the real echo, an attacker can spoof a distance that is shorter than the actual distance [6,9]. One major limitation of this method is the need to know the exact time of the transmitted pulse and the exact distance to the sensor, both of which are difficult to obtain, thus making this attack highly unlikely in some cases. One possible way to obtain the time of transmitted pulses is to measure the time between pulses and use this measurement to predict the time of the next pulse [9]; however, by design implementation or as a countermeasure, the sensor may introduce jitter to the timing or use asynchronous cycles [11], and at best, the attack can produce spoofed objects at random distances.
**Acoustic cancellation.** Acoustic cancellation, also known as active noise cancellation, is performed by transmitting a wave with a 180-degree phase on the receiver end. In this case, the amplitude of the echo will be decreased, and the sensor will not receive an echo signal [12]. Active noise cancellation is commonly used in headphones to mask environmental noise [13]. In an acoustic cancellation attack against the ultrasonic distance sensor, the adversary has to know the exact distance from the sensor to the object and the exact phase of the transmitter at the moment of the pulse creation. Such precise knowledge is extremely difficult to obtain and use during the attack; for example, assuming a typical 40 kHz ultrasonic signal, the wavelength is 8.5 mm. A drift of millimeters in the position of one of these three elements (attacker, sensor, or object) will result in an unsuccessful attack.
**Adversarial machine learning.** Manipulating the data (signal) that the sensor is measuring can have a negative impact on the autonomous vehicle and may result in a collision with a nearby object that was not detected. In [10], it was shown that other components that use the ultrasonic distance sensor reading can be affected by such an attack as well. An autonomous vehicle that uses a machine learning algorithm relies heavily on the sensor measurements; thus, an adversarial learning attack that manipulates the sensor readings (in the digital or physical domain) can severely affect the algorithm that governs the vehicle [10].

While relatively few studies have been dedicated to ultrasonic sensor attacks, they have recently received more attention in a broader context of attacks on sensors of autonomous vehicles and cyber-physical systems (surveyed in [14]). On the defense side, Xu et al. [9] suggested using signal randomization to thwart spoofing attacks. More recently, Lee et al. [15] proposed a mathematical model-based method for detecting signal injection attacks on ultrasonic sensors. While these methods are specific to ultrasonic sensors, they can also be defended against by using more generic approaches, such as sensor fusion, as suggested by Ivanov et al. in [16], applied to ultrasonic sensors in [9], and recently implemented by [17] in the context of spoofing attacks on related position sensors in autonomous vehicles. While, in this research, we consider an attacker that manipulates the physical environment, there are other attack vectors on ultrasonic sensors, such as replacing the sensor with a faulty or manipulated one or replaying its measurements using cyber attacks. To combat such attacks, Ahmed et al. [18] proposed a fingerprinting-based method and demonstrated it on ultrasonic sensors in a water treatment testbed. Similarly, Shoukry et al. [19] proposed detecting an adversary attack on active sensors by modulating a probing signal that continuously monitors the environment.

In this research, we present and evaluate a new and simple-to-implement spoofing attack that enables an attacker to control the readings of the sensor by changing the amplitude or frequency of a continuous sound signal generated by the attacker. Unlike previous attacks, the proposed attack is easy to implement and can be executed using inexpensive components. In addition, the attack is more difficult to detect than previous attacks, and in some scenarios, it can be effective against several targets at the same time. The attack is also resistant to jamming detection methods that look for abnormal changes in signal readings, since it is based on a continuous sound wave that can gradually change the distance perceived by the sensor.

## 3. Attack Description

In this section, we present the proposed spoofing attack (Section 3.1). Next, in Section 3.2, we describe the architecture of a common ultrasonic sensor used in autonomous vehicles and explain the behavior that we found allows the attack (i.e., the root cause of the attack). Section 3.3, presents an analysis and calculation for the number of transmitters required to attack a sensor at a desired distance. Finally, in Section 3.4, we present the properties of our attack.

### 3.1. Overview

In this section, we present the proposed spoofing attack on the UDS of an autonomous vehicle. The main goal is to implement an attack that allows the attacker to control and change the distance that is perceived/measured by the sensor. By doing so, an attacker can cause a malfunction in the vehicle’s proper operation, potentially causing a crash, or manipulate the vehicle’s behavior in a specific way for other purposes. In addition, the attack can be implemented as a countermeasure for preventing unwanted drones or robots from approaching sensitive facilities. This can be done by creating a “virtual wall” that prevents the vehicle from passing through.

**Figure 1:**
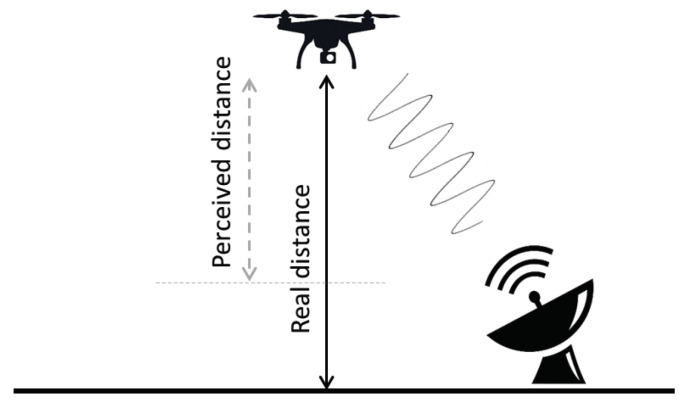
The proposed attack on the ultrasonic sensor using a continuous wave.

In our attack, using an ultrasonic transmitter positioned in the general direction of the sensor, we generate a *continuous wave* (see an illustration of this in Figure 1) that results in spoofing the real distance of objects. Since we use a continuous wave, there is no need to know the ultrasonic sensor’s pulse timing.

The signal strength (i.e., the continuous wave’s magnitude) generated by the attacker should be determined based on the desired change in the spoofed distance and the distance between the attacker and the sensor. The strength can be controlled by changing (1) the amplitude of the signal or (2) the frequency. The reason that a change in frequency results in changing the signal strength received by the sensor is due to the resonant frequency of the receiver. The receiver of an ultrasonic sensor uses a microphone that “listens” to a specific frequency. In order to operate it optimally, the resonant frequency of the receiver is set to be the same as the transmitting frequency. Any signal outside of the range of the resonant frequency will experience gradual damping.

### 3.2. Root Cause Analysis

**Figure 2:**
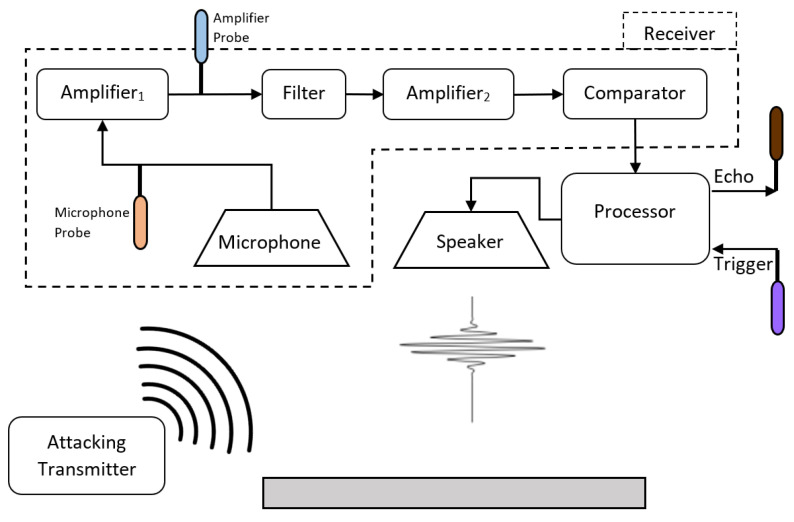
Sensor architecture and attack location.

**Figure 3:**
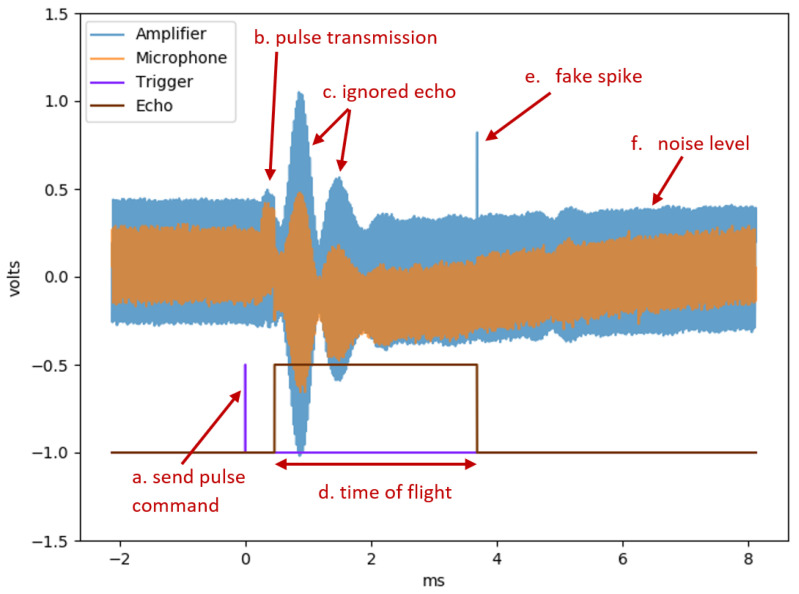
The result of monitoring the microphone (receiver) and the first amplifier ($amplifier_{1}$), which is connected directly to the microphone’s output, as the attack is in progress and a fake object is detected at a distance of 55 cm (when there was no real object within the sensor’s range).

Figure 2 presents the architecture of a common ultrasonic sensor used in autonomous vehicles and its signal processing. In order to understand the root cause of the attack, we probed the output of $amplifier_{1}$ and the microphone. Figure 3 presents the two analog signals of the microphone and $amplifier_{1}$ and two digital signals that represent the sensor control (trigger) and state (echo). The transmitter receives a trigger signal ((a) in Figure 3), which commands the transmitter to send a pulse. The transmitter sends a short pulse at the sensor’s working frequency in the direction of the scanned environment ((b) in Figure 3). After each pulse, the sensor waits for a fixed (predefined) time before listening to the returning echo ((c) in Figure 3) in order to eliminate noise produced by the transmitter and sensor hardware. The receiver listens to the returning echo, and at the time of detection, it changes the echo’s digital state (indicated by the falling edge of (d) in Figure 3). The received signal is processed by several components. The first amplifier ($amplifier_{1}$) increases the microphone’s signal, the band-pass filter cleans the signal (i.e., removes non-working frequencies), and after another amplification, a comparator is used to determine whether the signal is strong enough to be considered a valid echo. The processor orchestrates the object detection process, including controlling the transmitter, determining the duration of noises ignored, and validating the echos.

The attack is possible due to a phenomenon observed in the receiver amplifier when a constant wave is transmitted towards it. We observed an anomaly in amplifier1’s output in the form of a short spike ((d) in Figure 3). The microphone’s output contains no anomalies; therefore, we conclude that the root cause of the spike effect is produced by the analog components of the amplifier. Note that we eliminate the hypothesis that the anomaly is a result of weak echos from the environment by testing the effect in an isolated environment.

This anomaly of $amplifier_{1}$ results in a fake echo detected by the sensor. The echo’s digital signal ((d) in Figure 3) is the output of the sensor, and the falling edge indicates the time of the returning echo. The timing of the anomaly (at (e)) is aligned with the timing of the fake echo (at (d)). To summarize, we detect a fake pulse in the output of the first amplifier, which causes the sensor to detect it as a real echo.

Mathematically, the sensor calculates the distance to an object by the following equation [15]:

(1) d=(t/2)∗v

where *d* is the distance to the object, *t* is the amount of time between the original signal transmission and the returning echo registration, and *v* is the sound speed in the air, which is 343 m per second. As the sound travels from the sensor to the object and back, we divide the time *t* by two. With our attack, the fake spike (indicated by (e) in Figure 3) is mistakenly interpreted by the sensor as the returning echo, thus shortening the value of *t* and resulting in a short spoofed distance measured (i.e., a closer object). According to the classification by Lee et al. [15], we use an “ignorant attack” in which the attacker has no knowledge about the transmitting timing of the sensor but still achieves the effect of a “knowledgeable attack” in which such knowledge is needed to transmit a pulse at a particular moment in order to spoof the desired distance.

A change in the attacking signal strength results in a change in the timing of the spike (indicated by (e)). A stronger signal causes both the spike to appear earlier and the sensor to identify a closer fake object. A possible explanation for this is that charging capacitors change the timing of the spike. During the sensor’s pulse transmission, a lot of power is directed to the transmitter. This results in a temporary voltage drop across the receiver component, including the capacitor, which is part of the amplifier circuit. A noise can charge the capacitor at different rates. In an analog signal, circuits of the capacitors usually operate while partially charged. From the time that the circuit is started, the capacitors have to stabilize their voltage. This process requires some time, and during that time, fluctuations and even voltage spikes (such as the one observed in Figure 3 at (e)) in the circuit are common.

### 3.3. Speaker Strength Estimation

The attack effectiveness depends on the transmitter’s ability to create the required air pressure near the attacked microphone. The simplest way to determine the maximum attack distance for a given transmitter without additional specialized equipment is empirical testing. We used this approach in our study. If the required attack distance exceeds the maximum one for the given transmitter, multiple transmitters pointing in the same direction can be used. To calculate the number of transmitters needed to attack a sensor from a given distance, we need to know the power that one transmitter can output and the distance from which one transmitter can attack a sensor. We use the following notation (W stands for watt; m stands for meter):

- P(W)—the power of a single transmitter;
- $I(\frac{W}{m^{2}})$—the intensity of a transmitter;
- $A(m^{2})$—area of the transmitted signal at a distance from the transmitter;
- x(m)—the maximum effective attack distance of one transmitter; and
- $x_{d}\left(m\right)$—the desired attack distance.

We want to determine the multiplication factor of the power *P* required to affect a sensor at a distance $x_{d}$. The total power crossing any sphere surrounding the source is calculated by the intensity *I* times the area *A*:

(2) P=AI

In our case, the speaker is directional, meaning that the power is only distributed over part of the sphere; this area on a sphere face is computed by

(3) $$A=2\pi \left(1-\cos\left(\theta /2\right)\right)r^{2}$$

where θ is the transmitter’s beam angle. The power will be

(4) $$P=2\pi \left(1-\cos\left(\theta /2\right)\right)x^{2}I$$

The power multiplication factor required to reach the same intensity of a single transmitter at a closer distance is computed as follows:

(5) $$P^{\prime }=2\pi \left(1-\cos\left(\theta /2\right)\right){x_{d}}^{2}I$$

$$\begin{array}{c} \frac{P^{\prime }}{2\pi \left(1-\cos\left(\theta /2\right)\right){x_{d}}^{2}}=\frac{P}{2\pi \left(1-\cos\left(\theta /2\right)\right)x^{2}}\\ \frac{P^{\prime }}{P}={\left(\frac{x_{d}}{x}\right)}^{2}\\ \frac{P^{\prime }}{P}=D^{2} \end{array}$$

For a distance that is *D* times the original distance, we will need $D^{2}$ transmitters. For example, if we have one transmitter that can attack a sensor at a distance of one meter, and we want to implement the same attack from a 10 m distance, 100 transmitters will be needed.

### 3.4. Attack Properties

Based on our analysis of the proposed attack (presented in the previous subsections), we identified the following main properties of the attack:

**Easy to implement.** The attack is based on basic hardware and electronic components: (i) an ultrasonic speaker/transmitter, (ii) an amplifier, and (iii) a processor, each of which is inexpensive and easy to assemble. Ultrasonic sensors operate at a specific frequency. Therefore, the attacking transmitter needs to be able to transmit within close range of the same frequency. For the amplifier, it is possible to use a simple circuit with constant physical properties. Such a wave amplifier circuit can be made, for example, from transistors and capacitors with a fixed value to match the frequency. To control the frequency and amplitude, a simple computer (processor) and a sound driver can be used with an amplifier. Sound drivers can operate at a 192 kHz transmitting rate, which is sufficient for matching the typical 40 kHz of the sensor, as it has to operate at least twice the desired frequency. Even a 48 kHz sound card can be manipulated to transmit at double the speed with the dual channels in stereo. The digital stereo driver sends a signal to each channel at a time, so combining the channels into one gives us double the signal.
**Digital stealthiness.** A typical spoofing attack that relies on the timing of transmission is relatively easy to detect. The defender can monitor the environmental signals and look for anomalies [20] in order to differentiate legitimate echo pulses from malicious ones. Examples of signal characteristics that can be used to detect the attack are frequency deviation (the attacker’s signal frequency will be different from the sensor frequency) and echo amplitude (repeated strong echos may indicate a possible attack [21]). On the other hand, a constant wave is harder to detect, as it can be considered background noise, which is much more common than timed pulses.
**Implementation independent.** Previous spoofing attack scenarios require a known pulse timing, e.g., to measure the time between two pulses. These timings are dependent on the implementation of the sensor driver and not on the module itself. Therefore, two similar sensors may have two different behaviors. In our attack, we do not need to have any knowledge on the implementation of a known sensor.

## 4. Experiments

We designed a set of experiments to demonstrate the attack in a testbed environment as well as in a real-world scenario using an off-the-shelf drone. In this subsection, we present the performance of the attack in a testbed environment, as well as on a setup involving a real drone. We also evaluate the attack transferability and robustness and its effectiveness for longer distances (based on our analysis in Section 3.3). Finally, we compare our attack with a pulse-timing spoofing attack.

### 4.1. Testbed Experiments

**Figure 4:**
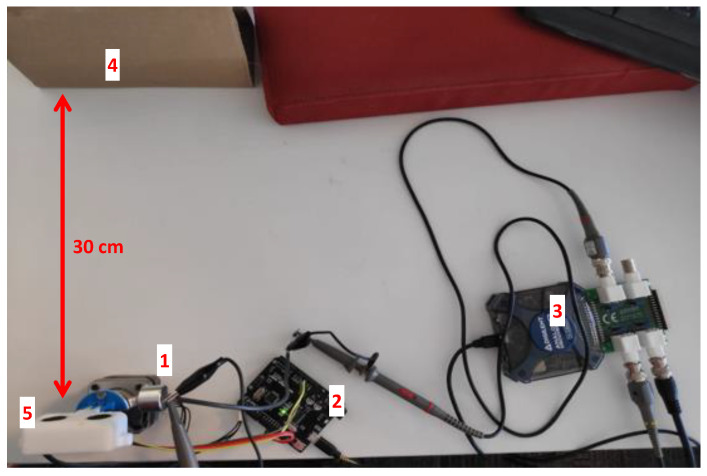
Experimental setup: 1—ultrasonic transmitter; 2—sensor controller; 3—function generator; 4—object; 5—sensor.

The testbed and its elements are presented in Figure 4. The experiment was set up in a small test environment made up of low-power components. We used multiple distance sensor modules ((5) in Figure 4), which are comprised of a transmitter, receiver, and driver. The sensor is controlled by an Arduino board ((2) in Figure 4) that triggers the sensor, instructs it to send a pulse, and measures the time elapsed until the sensor receives the return echo. The attacker setup consists of a transmitter ((1) in Figure 4) that is connected to an external sound card (Xonar U7 MKII). This external sound card acts as a function generator that is controlled by a PC. For the drone setup (presented in Section 4.5), we used an Analog Discovery 2 ((3) in Figure 4) as a function generator to produce a stronger signal. The Xonar U7 MKII sound card is capable of outputting data at a rate of 192 kHz, which is sufficient for transmitting a 40 kHz signal. The sensor faces a flat surface ((4) in Figure 4), which serves as the object, and the attacker’s transmitter is placed outside of the sensor’s field of operation, facing the sensor at a 30-degree angle.

In the experiment, the sensor operates normally. We consistently logged its readings while transmitting the constant signal using the attacker’s device (transmitter). We tested the setup using various attacking signal levels: (1) a constant signal amplitude with a changing frequency, (2) a constant frequency with a changing amplitude, and (3) various combinations using different signal frequencies and amplitude levels.

### 4.2. Attack Performance in the Testbed Environment

**Figure 5:**
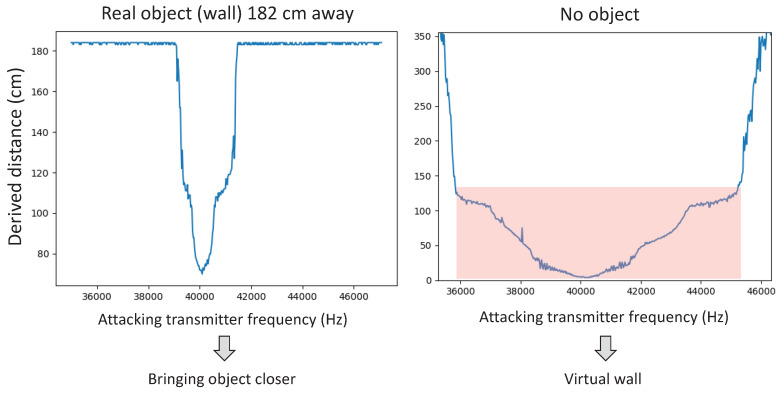
Transmitting a constant signal with a changing frequency.

Figure 5 shows how changing the frequency affects the distance measured by the sensor (HC-SR04). The graph on the left presents the sensor’s reading when the sensor is facing an object located 182 cm away. The graph on the right presents the sensor’s reading when no object is in the range of the sensor. Both graphs present the measured distance for various transmitted frequencies. Most noticeable in the figure is how the distance measured varies and is centered around the frequency of the sensor: the shortest distance to the object is measured around 40 kHz, and it increases when the frequency of the attacking signal increases or decreases.In the scenario in which the sensor is facing an object (left graph), the sensor derives the real distance from the object (182 cm) for frequencies that are ±1 kHz from the 40 kHz frequency. In the case in which the sensor is not facing an object (right graph), the normal sensor readings are “timed out,” as the sensor cannot see anything in its operational range. In this case, when the frequency of the attacker is 4–6 kHz away from the 40 kHz frequency (indicated by the shading in the right graph), the sensor mistakenly perceives an object facing the sensor at different distances, depending on the attacking signal frequency.

**Figure 6:**
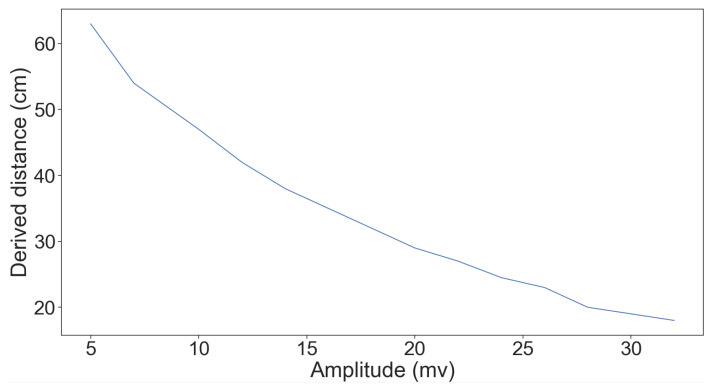
Changing the volume (attacker transmitter amplitudes) when the frequency is fixed (40 kHz). The sensor is 1.7 m from the object. In can be seen that as the strength increases, the perceived (measured) distance decreases.

Figure 6 shows the effect of changing the amplitude on the distance measured. We used the sound card that is connected to a PC in order to set the strength (amplitude) as a percentage of the card’s maximum output signal strength of 3.677 Vp-p (from 0 to 100%) when the frequency is fixed at 40 kHz and the object is 1.7 m away from the sensor. We can see an almost linear relation between the strength and the distance measured.

**Figure 7:**
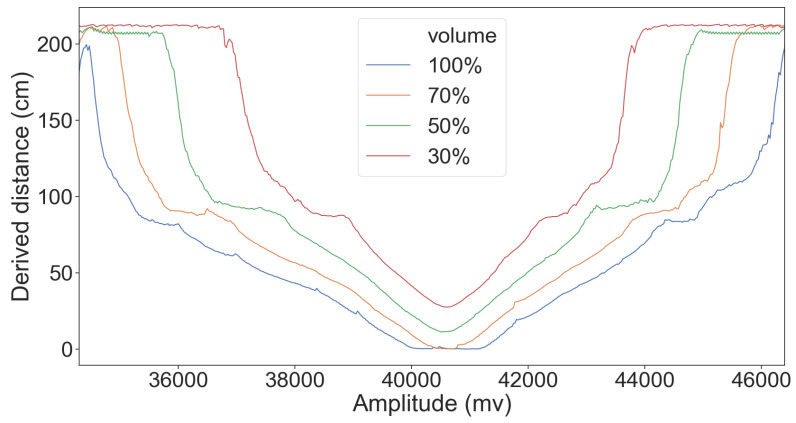
The effect of different combinations of attacker signal frequencies and strength on the sensor readings when there is no object in the sensor range.

Finally, in Figure 7, we present the distance derived for different frequencies when the amplitude is set at 60, 80, and 100% of the card’s maximum signal strength. Similar behavior can be observed for the different amplitudes, indicating that the attack is successful without special tuning of the attacker signal.

### 4.3. Attack Transferability

**Figure 8:**
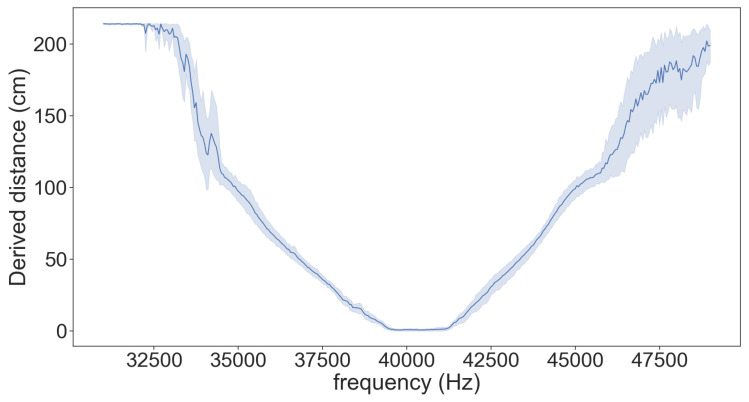
Evaluation of the transferability of the attack between different sensors of the same type. The graph presents the error deviation of the perceived distance when determining the attack parameters of one HC-SR04 sensor and then testing the attack on eight other HC-SR04 sensors.

In order to learn whether the attack is transferable and can be performed in a real-world scenario, we conducted the following experiment: we applied the attack on one sensor (HC-SR04) to determine the attack properties (i.e., the attack transmission frequency required for the desired spoofed distance), and then we repeated the attack with the derived parameters on eight other sensors of the same model (HC-SR04). The results presented in Figure 8 show that the attack is transferable between sensors of the same type.

### 4.4. Evaluating Different Sensor Models

In order to evaluate the robustness of the proposed attack, we tested the attack on 11 different ultrasonic sensor models. Different models use different hardware designs and components, and because the attack affects the analog part of the sensor, we expect different results for each model. We analyze and present the success of the attack according to the following two parameters: (i) Possible spoofing distance, which depends on the possible frequency range (this is represented, for example, by the frequency ranges from 32,500 to 40,000 in Figure 8); a steeper slope (i.e., narrow range of frequencies) results in a limited spoofing distance. (ii) The consistency of the change in the spoofed distance; in the optimal case, the change in the spoofed distance is monotonic with the changing frequencies.

**Figure 9:**
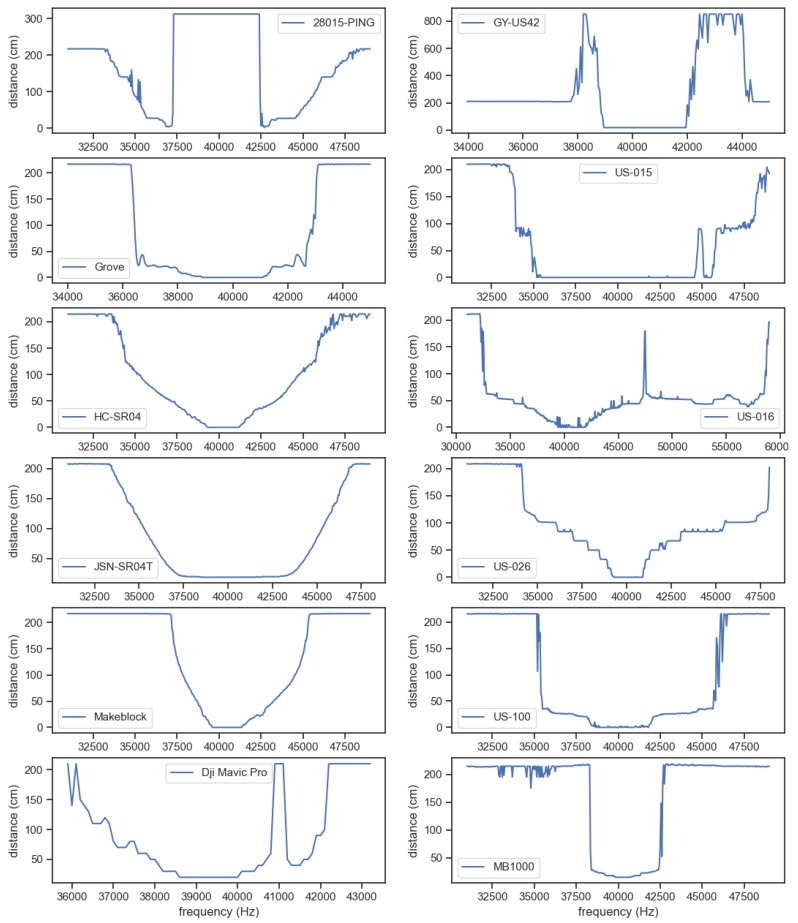
Graphs presenting the attack characteristics; i.e., distance measured by each of the 12 examined sensors for the different attacking signal frequencies. Each sensor can be identified by the label of each graph.

Table 2 presents the results of the attack on the different models evaluated. The results show that the attack is successful on 10 of the 11 sensors tested; in two cases (Grove and US-100), the attack is shown to be effective in a narrow frequency range (i.e., a short distance can be spoofed), and in three other cases (US-015, US-016, US-100), the attack is inconsistent at some frequencies. Figure 9 presents the attack characteristics (i.e., distance measured by the sensor for the different attacking signal frequencies) for all 12 of the sensors examined.

### 4.5. Real Setup Using a Drone

In order to demonstrate a scenario in which such an attack can be used on a real autonomous vehicle, we used a setup similar to the previously described testbed; however, instead of testing with a bare-bones sensor, we used a popular commercial drone—*DJI Mavic Pro*. The UDS used in the DJI Mavic Pro is a proprietary sensor implemented by the drone manufacture (DJI) and is soldered to the motherboard. For this experiment, we replaced the sound driver with a function generator, as it can produce the stronger signal required in this setup.

The drone is equipped with an ultrasonic sensor that is positioned on the underside of the drone. The sensor is used to determine the drone’s distance from the ground at low altitudes; this measurement is presented on the remote control. The sensor assists the drone, preventing it from hitting the ground when flying and helping it land safely. If the drone operator maneuvers the drone too close to the ground, the drone ignores a descend command in order to prevent a crash. When the drone operator hovers and wants to land the drone, he/she has to hold the elevation controller in the descend mode for a few moments, and then the drone safely lands by itself.

**Figure 10:**
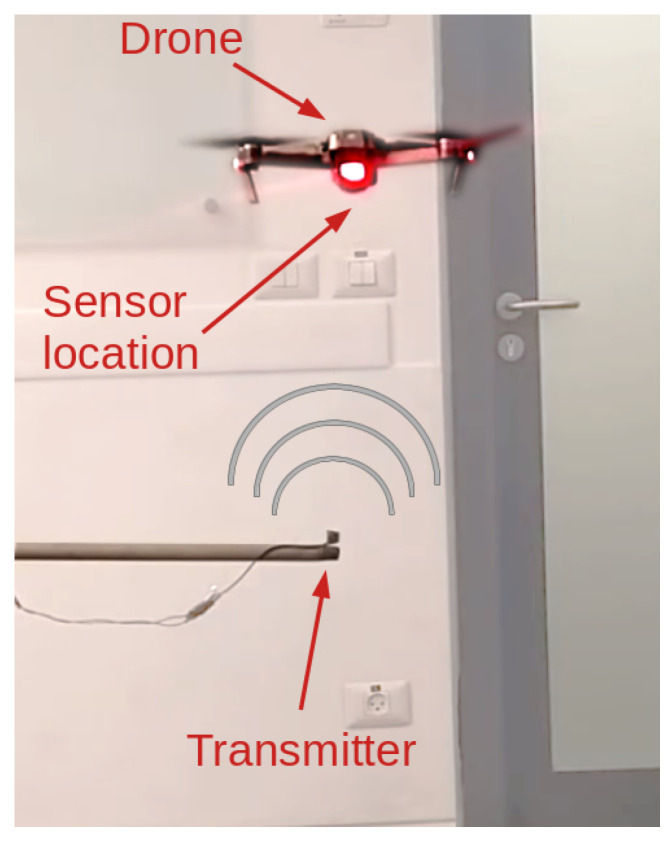
The real setup involving the drone. The drone is positioned above the transmitter. The transmitter is small enough so as not to be detected by the drone.

**Figure 11:**
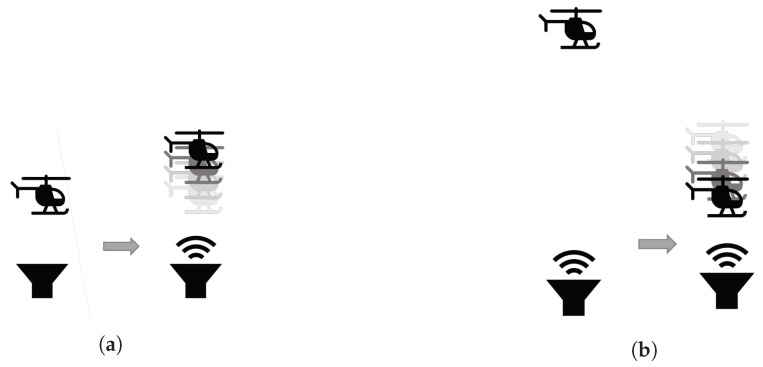
Two scenarios for attacks on a drone: (**a**) The drone hovers at a fixed distance from the ground when the attacker is not transmitting (i.e., the attacker’s transmitter is off). When the attacker starts transmitting, the drone gradually ascends and stops at a new height according to its new perceived distance from the “virtual” ground. (**b**) The attacker is transmitting the attacking signal, while the drone is commanded to descend. The drone stops at a preconfigured height from the attacking sensor according to the perceived distance from the “virtual” ground.

The test setup consists of a transmitter that is placed on a thin pole positioned above the ground and a drone that flies directly above it (see Figure 10). The drone’s sensor cannot detect the transmitter or the pole due to their small surface areas. The transmitter is connected by a thin wire to a function generator that is controlled by a computer. We conducted two experiments to examine two common scenarios involving drones. In the first experiment, the drone hovers at a constant height, and the attacker starts transmitting in the direction of the sensor https://youtu.be/nEsSkJuKIL4. At the moment of the attack, the drone gradually ascends until it stops at the perceived distance that it measured before the attack occurred, as illustrated in Figure 11a. In the second experiment, the attacker transmitter continuously transmits a constant wave upward towards the drone. The drone hovers above the transmitter at a height at which the attack does not affect the sensor (approximately one meter). At some point in time, the drone operator commands the drone to descend toward the transmitter https://youtu.be/178Uf5K6iC0. When the drone is in close proximity to the transmitter, it stops descending, as illustrated in Figure 11b. At that point, the operator stops the “descend command” to avoid damaging the drone. Note that holding the “descend command” for a long enough period of time will trigger the automatic landing procedure. In this case, the drone will attempt to land above ground level due to the attack.

**Figure 12:**
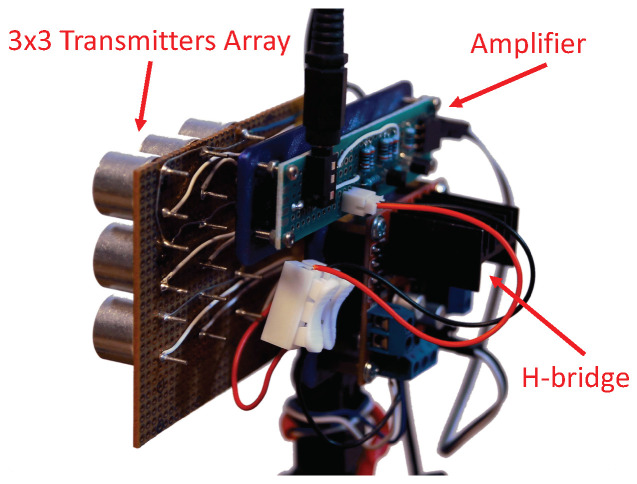
An extended setup of the attacking device composed of three elements: an amplifier, H-bridge, and nine transmitters in a 3 × 3 array. The device can receive a weak signal and amplify it using the transmitters.

Finally, to evaluate the effectiveness of the attack for longer distances (according to the analysis presented in Section 3.3), we implemented an array of nine transmitters in a 3 × 3 arrangement (as presented in Figure 12) and measured the distance for which the attack is effective for the DJI drone. Our tests show that the attack was effective for a distance of nine meters. This is aligned with our analysis presented in Section 3.3: the maximum attack distance of one transmitter is approximately three meters, and according to Equation (4), $3^{2}=9$. These experiments further validate the effectiveness of the proposed attack method against real-world vehicles.

### 4.6. Comparison with Pulse-Timing Spoofing Attack

In order to compare our attack with a classic UDS pulse-timing spoofing attack, we implemented the attack using the simple technique of transmitting a pulse at predetermined time intervals (as described in [9]). The time intervals should be determined for each specific object-to-sensor distance. We evaluated the attack on the HC-SR04 sensor. The HC-SR04 sensor operates according to the returning echo; that is, the time between two pulses sent by the sensor is relative to the distance measured to the closest object (i.e., the closer the object is, the higher the pulse frequency). Therefore, in order to implement the attack successfully, we had to determine the time that it takes for the sensor to transmit a pulse from the last received echo. With this knowledge, we could successfully apply a classic attack on the sensor and achieve similar performance to our proposed attack (see the first row in Table 2). It is important to note that the time period between two sent pulses is not determined by the sensor module but by the controller implementation (logic). Therefore, although both attacks (ours and the classic attack) obtained similar results on the HC-SR04 sensor, we claim that the advantage of our attack is twofold: First, the classic spoofing attack depends not only on the sensor model but also on the specific implementation of the controller and platform; this means that to successfully apply the attack, the attacker needs to know the exact platform (controller implementation) used, as well as the sensor model. Our attack, on the other hand, exploits a vulnerability in the sensor module itself and therefore is generic and platform/implementation independent. Second, in the classic attack implementation, the attacker must be able to measure the time between two pulses of the attacked sensor. Our proposed attack does not rely on any timing properties, and therefore, there is no need to listen to the sensor’s pulses.

### 4.7. Discussion

We demonstrate a novel spoofing attack on a UDS that is performed by transmitting a constant wave. Based on the experimental results, we conclude that the strength and frequency of the signal transmitted by the attacker determine the distance measured by the sensor.

The attacking module consists of commonly available components: a transmitter (like the TC40-16T) and a signal generator. The transmitter is made of an ultrasonic speaker and an amplifier, and the generator can be a simple microcontroller that will produce the pulse. This is possible due to the relatively low frequencies of ultrasound compared to today’s simple microcontrollers. The power consumed by one module is approximately 150 mW. The cost of the hardware for one attacking unit is comparable to the cost of a simple ultrasonic sensor (e.g., the HC-SR04, which costs around a dollar), as it shares most of its parts. Alternatively, the attacker can simply reuse the transmitter part of such a sensor. For controlling the module, a simple processor like ATtiny13A (which costs approximately a dollar) can be used. If the attacker requires a greater distance, then multiple transmitters should be combined in a setup similar to the one presented in Figure 12 (which can attack from a distance of nine meters). It should be mentioned that although ultrasonic sensors are limited to measuring distances up to a few meters by the sound attenuation in the air, the attacker in our setup is not limited to any distance, provided that he/she has enough transmitting power.

Despite the similarity between the results of our attack and those of a previously presented spoofing attack, the attacks differ in terms of the method used and the scenario in which they are implemented. In a spoofing attack, the attacker must know the exact distance to the sensor and the exact timing of the pulse sent by the sensor. In our attack, only the distance to the sensor must be known by the attacker in order to change the frequency or the amplitude and spoof a specific distance.

In the case of an approaching (moving) vehicle, the attacker can use the change in the strength of the signal received by the vehicle’s sensor to his/her advantage. If the attacker wants to spoof the existence of an object, he/she simply needs to place a transmitter at a fixed location and transmit a signal with constant strength (amplitude) according to the distance that he/she wants to simulate. As the vehicle approaches the transmitter of the attacker, it will gradually perceive a virtual object approaching at a high speed, as the attack signal’s strength increases as the vehicle gets closer to the attacker. One use case of such an attack method includes placing the spoofing transmitter on the roof of a sensitive facility to prevent drones from landing on or even approaching the facility. This use case was successfully demonstrated in the second experiment that was conducted using the commercial drone. Unlike the work of Dumitrescu et al. [22], who proposed an ultrasonic-based method for detecting unauthorized drones, our method can be a complementary, preventative one.

The attacker is required to be able to control the attacking signal’s properties (i.e., amplitude and frequency) based on both the distance to the sensor and the sensor’s operating frequency. These requirements of the proposed attack create the following three limitations: First, the attacker has to know the distance to the sensor. This is, however, also a requirement for the previously presented classic pulse timing attacks. Second, the frequency received on the sensor side can be altered by the Doppler effect in cases in which the target is changing its distance fast enough in relation to the attacker’s location. Third, because of the directionality of the sensor’s receiver, the attacker needs to transmit the attacking signal within the sensor’s receiver angle range; this is also a limitation of the classic spoofing attack.

## 5. Countermeasures

We identified the following possible countermeasures that can be applied to detect and/or prevent the proposed attack.

**Frequency hopping.** In this approach, commonly used to overcome jamming attacks, the sensor periodically and randomly changes the frequency of the transmitted signal. However, the use of frequency hopping will significantly increase the cost of the sensor; note that the main advantage of the UDS is its very low cost. In addition, as shown by the experimental results, the attack is robust and does not rely on identifying a specific frequency; thus, the attacker can transmit signals at several frequencies and cover a wide range of ultrasonic signals.
**Sensor fusion.** Sensor fusion correlates the conclusions derived from the sensor’s readings (i.e., distance to nearby objects) with other sensors’ data. For example, if a drone is hovering above the ground at a fixed height during the proposed attack, the UDS will perceive a change in the height, but the accelerometer sensor will not detect any movement. This approach increases the processing time, requires additional computation capabilities, and is vulnerable to false positives.
**Detecting strong noise in the operating frequency range.** Strong noise in the sensor’s operating frequency range can be detected by the vehicle using additional mechanisms and may indicate a possible attack. However, this technique only allows for detecting the attack, and it does not provide the means to mitigate it. Therefore, upon detection, the vehicle will not be able to use its USD, thus turning the spoofing attack into a denial-of-service attack.
**Machine learning.** Machine learning methods can be used to detect attack attempts [23,24]. By learning the patterns of echo signals, a fake pulse can be distinguished from a benign signal. This approach, however, requires additional computation capabilities, may suffer from false positives, and can only be used to detect the attack and will be unable to mitigate it.

In Table 3, we summarize the proposed countermeasures, along with the hardware and software changes required for implementing the countermeasures.

## 6. Conclusions and Future Work

In this paper, we present a spoofing attack on a UDS sensor that does not require precise knowledge on the sensor’s pulse timing. To the best of our knowledge, this type of attack has not been demonstrated before. Spoofing is one of the most powerful attacks aimed at UDSs, but it is usually very difficult to implement and even harder to execute in a real environment. Our approach enables the execution of a spoofing attack with a much simpler setup. Cost is also a major advantage of the proposed attack: the total cost of our setup is less and the equipment required is more readily available than those of a classic spoofing attack.

Another relevant scenario in which our method can be used is to defend against unauthorized drones at sensitive facilities. Such facilities are often the target of espionage or sabotage, leaving them vulnerable to drones that approach the premises for those purposes. To protect a surface from drone landings and protect such facilities, a defensive device based on our method can be placed on the roof of the building (with the transmitter facing up); any drone that attempts to land will perceive the ground as being closer than it really is, and this will prevent the drone from descending beyond a certain limit or cause it to crash.

With a constant wave attack, where a signal is transmitted at a fixed amplitude and frequency, it is possible to spoof a specific distance. To determine the exact spoofing distance, the attacker needs to know his/her distance to the vehicle (sensor). Despite the attack’s simplicity and effectiveness, two factors can limit its use for spoofing objects that are far away: the difficulty in transmitting sound over long distances and the transmitting power available to the attacker.

In terms of defense mechanisms aimed at countering the attack, there are a few routes to be considered, including developing new hardware designs that can prevent the effect that facilitates the attack; using software that can analyze the sensor data, detect attack attempts, and possibly remediate it; and developing a mechanism for monitoring the environment for signals that match the attacking signal and disabling it before a vehicle is in the vicinity.

We believe that this paper, in which we demonstrate this novel sensor’s vulnerability, will increase awareness in manufacturers and users and stimulate further research that results in additional use cases for such attacks.

## Figures and Tables

**Table 1:** Summary of previous work related to attacks on ultrasonic distance sensors as well as our proposed attack.

Attack Category [Reference]	Goal	Method
Denial-of-service (jamming) [6,8]	Making the sensor unusable by presenting fixed or random values	Producing an external interference (noise) that the sensor will receive as the returned echo
Spoofing [6,9]	Faking an object; the fake object is a completely new object unrelated to the environment in a specific or random location	Transmitting a pulse of an ultrasonic wave at a specific time that the sensor will perceive as a legitimate echo; the timing of the pulse will determine the location of the fake object
Acoustic cancellation [10]	Hiding objects (making an object transparent)	Transmitting a signal with the exact frequencies and phase so that the real signal of the sensor will interact with the attacker’s signal, and they will cancel each other
Adversarial machine learning attack [10]	Causing abnormal behavior of a vehicle that is controlled by a machine learning algorithm	Applying an adversarial learning attack by manipulating the sensor readings in the physical or digital domain
Our proposed spoofing attack	Affecting the readings of the sensor by using a continuous sound signal generated by an attacker and disrupting the proper operation of the vehicle	Transmitting a constant signal to affect a sensor; changing the amplitude/frequency to control the sensor readings

**Table 2:** The results of the attack on different sensor models. We present the sensor model, the frequency at which the sensor operates, the type of the sensor (transmitter/receiver or transducer), an indication of whether the attack was successful or not, the spoofing distance, and finally, whether the attack is consistent (i.e., monotonic).

Sensor Model	Sensor Frequency	Sensor Type	Attack’s Success	Effective Frequency Range (Spoofing Distance)	Consistency
HC-SR04	40 kHz	transmitter receiver	Yes	wide	consistent
28015-PING	40 kHz	transmitter receiver	Yes	wide	consistent
Grove	40 kHz	transmitter receiver	Yes	narrow	consistent
JSN-SR04T	40 kHz	transducer	Yes	wide	consistent
Makeblock	40 kHz	transmitter receiver	Yes	wide	consistent
MB1000	41 kHz	transducer	No	-	-
US-015	40 kHz	transmitter receiver	Yes	wide	inconsistent
US-016	41 kHz	transmitter receiver	Yes	wide	inconsistent
US-026	40 kHz	transmitter receiver	Yes	wide	consistent
US-100	40 kHz	transmitter receiver	Yes	narrow	inconsistent
GY-US42	40 kHz	transmitter receiver	Yes	wide	consistent
DJI Mavic Pro (real setup)	40 kHz	transmitter receiver	Yes	wide	mostly consistent

**Table 3:** Proposed countermeasures, along with the hardware and software changes required for implementation.

Countermeasure	Mitigation Approach	Hardware Changes Required	Software Changes Required
Frequency hopping	Increasing sensor’s robustness	Significantly more complex sensor	Changes in sensor’s driver
Sensor fusion	Increasing sensor’s redundancy; Environment monitoring	Additional and redundant sensors	Algorithms for sensor data fusion
Adaptive noise sensing	Environment monitoring	Additional sensor capabilities	None
Signal anomaly detection (by ML and other methods)	Intelligent signal processing	Might require changes to the sensor to output additional signal information	Anomaly detection algorithms
